# The effects of exercise on the quality of life of patients with breast cancer (the UMBRELLA Fit study): study protocol for a randomized controlled trial

**DOI:** 10.1186/s13063-017-2252-5

**Published:** 2017-10-27

**Authors:** Roxanne Gal, Evelyn M. Monninkhof, Rolf H. H. Groenwold, Carla H. van Gils, Desiree H. J. G. van den Bongard, Petra H. M. Peeters, Helena M. Verkooijen, Anne M. May

**Affiliations:** 1 Department of Clinical Epidemiology, Julius Center for Health Sciences and Primary Care, University Medical Center Utrecht, Utrecht, The Netherlands; 2 Department of Radiation Oncology, University Medical Centre Utrecht, Utrecht, The Netherlands; 3 Imaging Division, University Medical Centre Utrecht, Utrecht, The Netherlands

**Keywords:** Breast cancer, Physical activity, Exercise, Cohort multiple randomized controlled trial, Quality of life

## Abstract

**Background:**

Meta-analyses of randomized controlled trials (RCTs) have shown that exercise has beneficial effects on quality of life (QoL) in patients with breast cancer. However, these effects were often small. Blinding in an exercise trial is not possible, which has the possible disadvantage of difficult accrual, drop-out after randomization to control and contamination between study groups (controls adopting the behaviour of the intervention group). The cohort multiple randomized controlled trial (cmRCT) is an alternative for conventional RCTs and has the potential to overcome these disadvantages.

**Methods:**

This cmRCT will be performed within the Utrecht cohort for Multiple BREast cancer intervention studies and Long-term evaLuAtion (UMBRELLA). Patients with breast cancer who visit the radiotherapy department of the University Medical Center Utrecht are asked to participate in UMBRELLA. Patients give consent for collection of medical information, providing patient-reported outcomes through regular questionnaires and randomization into future intervention studies. Patients who fulfill the UMBRELLA Fit study eligibility criteria (12 to 18 months post inclusion in UMBRELLA, low physical activity level) will be randomly allocated to the intervention or control group (1:1 ratio). Patients randomized to the intervention group will be offered a 12-week exercise programme. The control group will not be informed. Regular cohort measurements will be used for outcome assessment. Feasiblity (including participation, contamination, generalizability and retention) of the cmRCT design and effects of the intervention on QoL will be evaluated.

**Discussion:**

We will examine the feasibility of the cmRCT design in exercise-oncology research and compare this with conventional RCTs. Furthermore, the effectiveness of an exercise intervention on the QoL of patients with breast cancer in the short term (6 months) and long term (24 months) will be studied.

**Trial registration:**

Netherlands Trial Register, NTR5482/NL.52062.041.15. Retrospectively registered on 7 December 2015.

**Electronic supplementary material:**

The online version of this article (doi:10.1186/s13063-017-2252-5) contains supplementary material, which is available to authorized users.

## Background

Breast cancer is the most common cancer in women worldwide [1]. In developed countries, more than 85% of women survive for 5 years or longer [2]. As a result, many women live with the consequences of the disease and its treatment. Most patients with breast cancer experience side effects of treatment, including fatigue and impaired quality of life (QoL) [3–6].

Meta-analyses of randomized controlled trials (RCTs) have shown that exercise has beneficial effects on fatigue and QoL in patients with breast cancer [7–10]. Positive effects of physical activity on survival and recurrence have been found in observational research [11]. On the contrary, more sedentary behaviour is associated with higher fatigue levels [12], especially in patients with breast cancer with lower levels of physical activity [13, 14]. Reported exercise effects on fatigue and QoL are often small and this might be partly explained by shortcomings of the RCT design in evaluating exercise interventions that cannot be blinded [15]. The possible disadvantage of not blinding is contamination between study groups, high drop-out after randomization to the control group and difficult accrual [16–18].

The cohort multiple randomized controlled trial (cmRCT) design has been proposed as an alternative to conventional (pragmatic) RCTs [19]. In a cmRCT, the intervention study is performed within a large observational cohort with regular outcome measurements. At cohort entry, two-stage consent is requested for participation in the cohort study and to be randomized for future intervention studies [20]. After randomization, patients who are allocated to the intervention group will be offered the intervention. The control group will not be informed about their role as control in the exercise trial. Outcomes of the intervention group are compared to the outcomes of the control group, using the regular cohort measurements.

### Relative merits of the cmRCT design

The cmRCT design has the potential to overcome some challenges faced in (pragmatic) RCTs studying effects of exercise interventions in patients with cancer. First, in exercise-oncology research, blinding is impossible. As a result, participants allocated to the control group will often also increase their physical activity after randomization [16]. Substantial cross-over of control participants has occurred in over 35% of exercise-oncology RCTs, which might have led to dilution of the intervention effects [16]. Patients with cancer in particular are often motivated to change their lifestyle after diagnosis and, therefore, already intend to increase physical activity levels or attend an exercise programme in the near future [21]. For example, in the Physical Activity during Cancer Treatment (PACT) study, about 50% of the patients with breast cancer randomized to the control group adopted the behaviour of the intervention group [22]. A high level of contamination due to cross-over of controls who adopt the behaviour of the intervention group may decrease the statistical power to detect an intervention effect. Since the control group will not be informed in the cmRCT design about their role as control in the exercise trial, the risk of contamination will be reduced as a result of study participation [23]. Any increase of physical activity levels in the control group now reflects real life and is not caused by participation in an exercise RCT. Furthermore, drop-out in the control group due to disappointment in their allocation will not occur [16, 17].

Previous research has shown that patients with breast cancer who refuse participation in an exercise intervention study are on average older, more fatigued and had more comorbidities than trial participants [18, 24]. Hence, the study population is in general a selective group of younger or less fatigued patients with breast cancer or patients who had less comorbidity after treatment. In addition, higher-educated patients are more willing to participate in health behavior-change interventions [13, 24]. Reasons for the selection may be that trial participation is (unconsciously) not offered to specific subgroups (although fitting the eligibility criteria), that these patients are not willing to participate in an RCT with a 50% chance to be randomized to control or that patients do not want to exercise. Consequently, the generalizability of results will be reduced. Since in a cmRCT design the intervention is offered to eligible patients after randomization and no decisions on being randomized need to be made, participation might be less selective and reflect the real world. As a result, generalizability will increase. However, a prerequisite is that the cohort is representative for the study domain.

Also, long-term effects of physical activity on QoL and fatigue are not clear yet. The cmRCT design is a useful design to study effects in the long term because regular measurements in the prospective cohort will be continued after completion of the exercise intervention trial.

Finally, we expect that recruitment of participants will be easier and faster because patients will be recruited from the cohort. Data obtained by routine measurements can be used to identify eligible patients for the specific trial. Since patients have already signed informed consent to be randomized for future intervention studies, eligible patients allocated to the intervention group can directly be invited by the researchers. Hence, it is not necessary to set up trial recruitment logistics and to recruit a new group of patients with breast cancer.

In addition to the potential benefits, we also expect some challenges. In the cmRCT design, the intervention group is invited for participation after randomization and subsequently do not have to decide about participation in a randomized study. They only need to decide whether they accept or refuse the invitation of an exercise intervention. It might be that due to this approach less motivated patients may also participate, which might result in more drop-out in the intervention group than in the (non-informed) control group. Moreover, more patients with comorbidities might participate in the study compared to conventional RCTs, which also increases the risk of drop-out. Dependent on the amount of drop-out, this will impact the effect estimates from the intention-to-treat analyses [23].

In the UMBRELLA Fit study, the effect of a 12-week supervised exercise intervention on the QoL of patients with breast cancer in the short term and long term will be studied, using the cmRCT design.

## Methods/design

### Aim of this study

The UMBRELLA Fit study follows the cmRCT design (see Additional file 1). Here, patients with breast cancer who participate in the UMBRELLA breast cancer cohort (Utrecht cohort for Multiple BReast cancer intErvention studies and Long-term evaLuAtion) will be randomly selected for a 12-week supervised exercise intervention, 12 or 18 months after enrollment (i.e. postoperatively, prior to consultation of the radiation oncologist) in the UMBRELLA cohort. The purpose of the UMBRELLA Fit study is twofold. The first aim is to assess the feasibility and therefore, we will (1) assess the participation rate and intervention compliance in the intervention group and drop-out rates in the intervention and control group; (2) compare the (change in) physical activity levels in the intervention and control group during the intervention period; and (3) assess whether a more diverse generalizable study population will be included by comparing characteristics of the included study population with characteristics of patients participating in previous breast cancer exercise trials with a conventional RCT design. Our second goal is to examine the effectiveness of the exercise intervention on the QoL of patients with breast cancer in the short term (6 months) and long term (24 months).

### Patients

**Fig. 1:**
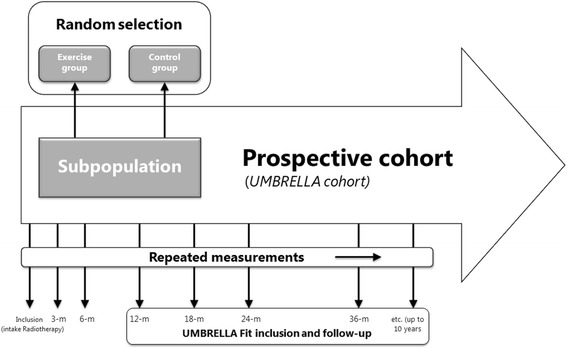
The cohort multiple randomized controlled trial (cmRCT) design (adapted from Relton et al.)

**Fig. 2:**
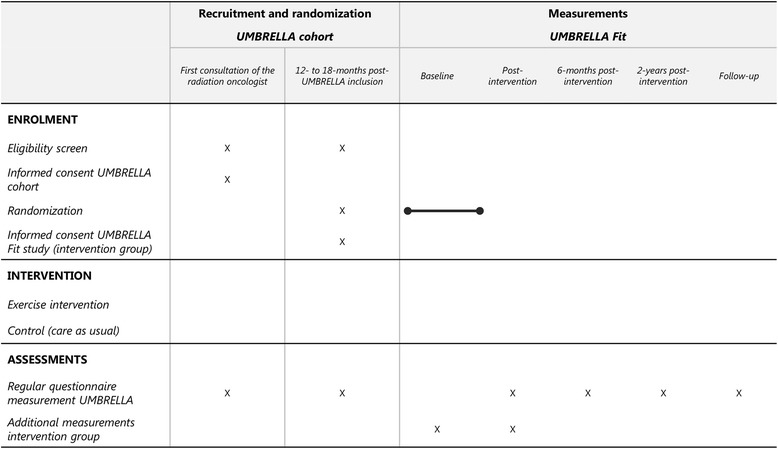
Standard Protocol Items for Intervention Trials (SPIRIT): schedule of enrollment, interventions and assessments

We will make use of the UMBRELLA cohort, which aims to generate short-term and long-term data on clinical and patient-reported outcomes during and after breast cancer treatment and to provide infrastructure for multiple randomized evaluations of the intervention in patients with breast cancer [25]. Recruitment started in September 2013 at the University Medical Centre (UMC) Utrecht and all patients with breast cancer who are referred to the department of radiation oncology of the UMC Utrecht for radiation treatment are asked to participate in the UMBRELLA (see Figs. 1 and 2) [25]. They give consent for the use of routinely collected clinical data and prospective collection of patient-reported outcome measures through regular questionnaires. In addition, they can opt to give consent to be randomized for future intervention studies within the cohort. Patients will be informed that they will be offered an experimental intervention if they are randomly selected for the intervention group. They are also informed that they will not be contacted when randomly selected for the control group and that their data can be used in a trial context [20]. Between the start of recruitment and July 2016, 88% of the eligible patients who were invited for participation in UMBRELLA gave consent to participate and 87% of those who agreed to participate also consented to be randomized for future intervention studies within the cohort [25].

For the UMBRELLA Fit study, participants in UMBRELLA who meet the following criteria are eligible: (1) UMBRELLA informed consent for randomization to future intervention studies; (2) 18–75 years of age; (3) 12 months or 18 months after enrollment in the UMBRELLA cohort, (4) primary cancer treatment completed (except for adjuvant hormonal treatment); and (5) a physically inactive lifestyle (less than 150 min/week performing moderate to vigorous leisure time and sports activities, ≥ 4 metabolic equivalent (MET)) as measured by the Short QUestionnaire to ASsess Health-enhancing physical activity (SQUASH) [26]. Patients with a contra-indication (e.g. neurological problems, arrhythmias and walking problems) to exercise will be excluded.

### Recruitment and allocation

UMBRELLA participants meeting the inclusion criteria will be randomly allocated to the intervention or control group with a 1:1 ratio, by an independent data manager using a computer-generated randomization list. Randomization will be stratified for time since enrollment (12 or 18 months). Patients randomized to the intervention group will receive an offer by mail to participate in an exercise programme, a patient information sheet and an informed consent form. After 1–2 weeks these patients are contacted by telephone to further explain the study. If patients decide to participate, a last check of eligibility will be performed. Contra-indications to the exercise programme will be screened using the Physical Activity Readiness Questionnaire (PAR-Q) [27]. If eligible, they will be invited to visit the UMC Utrecht to sign the informed consent form for the UMBRELLA Fit study and undertake a baseline assessment. If patients refuse to participate because of bad timing of the invitation, they will be asked if they are willing to start later. Patients randomized to the control group will not be informed about the study. Recruitment started in October 2015.

### Exercise intervention

The exercise intervention is a 12-week structured exercise programme. Patients are offered two one-hour supervised fitness group or individual sessions per week at a physiotherapist centre close to the patient’s home. The training programme is a combination of moderate to high-intensity aerobic training and strength training. Sessions include a 5-minute warm up, aerobic and strength training (50 minutes) and a cool down.

The programme will be tailored to the patient’s physical fitness level. The intensity of aerobic training is determined by the heart rate reserve, based on the guidelines of the American College of Sports Medicine [28]. Patients’ individualized target heart rate zones during aerobic training are based on the results of a cardiopulmonary exercise test which is performed at baseline. Target hearts rates are calculated by the formula:

$$ \mathrm{Target}\kern0.17em \mathrm{heart}\kern0.17em \mathrm{rate}=\mathrm{resting}\kern0.17em \mathrm{heart}\kern0.17em \mathrm{rate}+\left[\mathrm{intensity}{\left(\%\right)}^{\ast}\left(\mathrm{maximal}\kern0.17em \mathrm{heart}\kern0.17em \mathrm{rate}-\mathrm{resting}\kern0.17em \mathrm{heart}\kern0.17em \mathrm{rate}\right)\right] $$

Aerobic training will be performed on an exercise machine (e.g. a treadmill, exercise bike, crosstrainer), depending on the patient’s preference. The intensity and duration of the aerobic training will be gradually increased (Table 1).

**Table 1:** Overview of the exercise programme

Week	Aerobic training	Strength training
1–3	15–20 min 40–60% HRR + HR_rest_	1 set of 20–25 repetitions (20 RM test) of 9 exercises: row (back), chest press (chest), squat (legs), shoulder press (shoulders), bicep curl (biceps), lunges (legs), calf raises (calves), tricep extension (triceps), crunch (abdominals, 20–40 repetitions)
4–8	15–20 min 60–70% HRR + HR_rest_ 5–10 min 70–89% HRR + HR_rest_	2 sets of 15–20 repetitions (15 RM test) of 7 exercises: row (back), chest press (chest), squat (legs, 20–25 repetitions), shoulder press (shoulders, 10–15 repetitions), bicep curl (biceps), tricep extension (triceps), crunch or hoover (abdominals, 30–50 repetitions or seconds)
9–12	10 min 60–75% HRR + HR_rest_ Interval training: 10 × 30 sec, 1 min active rest between each interval	

*HRR*: heart rate reserve, *HR*_*rest*_: resting heart rate

HRR = maximal heart rate - resting heart rate

Strength training consists of a set of exercises, focusing on all major muscle groups. During the intake session at the physiotherapist centre, 20 repetition maximum (RM) tests will be performed to determine the training load (the maximum weight at which a muscle group can perform 20 repetitions) for the different exercises. These weights will be the starting point for each strength exercise. A 20 RM and 15 RM test will be repeated in the fourth and eight weeks of the programme (Table 1). In the case of physical limitations, the exercise programme will be adapted in consultation with the researchers.

In addition to the supervised exercise programme, patients will be stimulated to develop an active lifestyle. An active lifestyle is defined as being moderate to highly physically active for at least 30 min a day, according to the World Cancer Research Fund/AICR, American Institute for Cancer Research (WCRF/AICR) guidelines for patients with cancer (www.aicr.org). If necessary, in consideration with the physiotherapist, patients will gradually increase their physical activity level during the first four weeks of the programme until they adhere to the WCRF/AICR guideline.

Patients will also be encouraged to reduce sedentary time during the day. Patients will receive a wristband, the Jawbone UP2™ (Jawbone, San Francisco, CA, USA), to track activity and raise awareness of their sedentary behaviour. The “Idle” alert will be used, i.e. the band gently vibrates when the patient has been inactive for a period of 45 minutes. Furthermore, the activity tracker and the accompanying Smart Coach application will be used to encourage an active lifestyle by, amongst others, setting personal step-goals per day and giving personalized feedback on patients’ activity levels.

To enhance adherence to the exercise programme and help patients to achieve and maintain an active lifestyle, physiotherapists are instructed to motivate patients according to the principles of Bandura’s social cognitive theory (SCT), i.e. enhancing self-efficacy by giving positive feedback on their progress and the starting point of the training schedule has been carefully chosen so it will be likely that the patient will succeed [29]. As a result, it is expected that patients develop a feeling of mastery (mastery experience).

### Control group

Control patients are not aware of study participation and will receive usual care. After completion of the UMBRELLA Fit study, all UMBRELLA participants, irrespective of participation in this study, will be informed by the annual newsletter.

### Endpoints

#### Methodological endpoints

##### Acceptance of the intervention

Acceptance of the intervention is defined as the percentage of patients who are randomized to the exercise intervention and accept participation.

##### Physical activity behaviour

To gain insight into the contrast between physical activity levels, changes in physical activity level in the control group will be described and compared to changes in the intervention group. Since the control group is not aware of study participation, a possible increase in physical activity level in the control group (contamination) is not the result of study participation but may be explained by real-life factors, e.g. motivation to change their lifestyle after diagnosis. Physical activity level will be evaluated in regular cohort measurements using the SQUASH [26]. This questionnaire contains questions on commuting activities, leisure-time and sports activities, household activities and activities at work. It consists of three main queries: days per week, average time per day and intensity.

##### Generalizability

To assess generalizability, socio-demographics (e.g. age, educational level and household status) and medical information (e.g. diagnosis, tumour type, disease stage, type of treatment and comorbidity) of the UMBRELLA Fit study population will be compared to characteristics of more than 3163 patients with breast cancer participating in exercise trials, available from the POLARIS database with individual patient data from 34 international exercise-oncology trials [10]. Socio-demographical information on the UMBRELLA Fit study population is registered in, and obtained from the UMBRELLA cohort. Medical information is obtained from the Netherlands Cancer Registry (NCR), which is managed by the Netherlands Comprehensive Cancer Organisation (IKNL).

##### Trial retention

Retention will be scored as the rate of drop-out in the intervention group after signing informed consent for the UMBRELLA Fit study.

##### Cohort retention

Drop-out in the intervention and control group will be defined as withdrawal from the UMBRELLA cohort and/or non-response to the regular cohort measurements and will be scored for both groups.

#### Effectiveness endpoints

We will use the regular questionnaire measurements of the UMBRELLA cohort to assess QoL, fatigue, anxiety and depression, and sedentary behaviour. Patients are included 12 or 18 months after enrollment in the UMBRELLA cohort. The most recent questionnaires filled in before enrollment in the UMBRELLA Fit study will serve as baseline measurements. Questionnaires 6 and 24 months later will be used as follow-up measurements.

##### Quality of life

The European Organisation for Research and Treatment of Cancer Quality of Life Questionnaire C30 (EORTC QLQ-C30) is a 30-item questionnaire to assess the quality of life of patients with cancer [30]. The questionnaire comprises five functional scales (physical, role, emotional, cognitive and social), three symptom scales (fatigue, pain and nausea/vomiting) and a global health status/QoL scale.

Sedentary time will be assessed by two questions from the IPAQ questionnaire estimating weekly sitting time [31].

##### Anxiety and depression

Anxiety and depression will be assessed using the validated Dutch language version of the Hospital Anxiety and Depression Scale (HADS) [32]. This questionnaire consists of seven items for the depression subscale and seven items for the anxiety subscale, resulting in a total depression score and a total anxiety score.

### Additional measurements for the patients in the intervention group

Patients in the intervention group will visit the UMC Utrecht for additional measurements at the start and end of the intervention. These measurements include anthropometrics, cardiopulmonary exercise testing (CPET) and questionnaires to assess QoL, fatigue and physical activity (also completed with the regular measurements in the UMBRELLA cohort).

#### Anthropometrics

Body weight and height will be measured while patients wear light clothes and no shoes. A digital balance is used to measure weight (to the nearest 0.1 kg) and wall-mounted tape to measure height (to the nearest 0.1 cm). Body fat distribution will be assessed by the waist and hip circumference (to the nearest 0.5 cm) in duplicate and averaged. Waist circumference is measured while standing, midway between the lower ribs and the iliac crest. Hip circumference is measured across the buttocks, while standing.

#### Cardiorespiratory fitness

To determine cardiorespiratory fitness, peak oxygen uptake (VO2_peak_) and anaerobic threshold will be assessed by a CPET using a cycle ergometer and a ramp protocol. Expired gases and minute ventilation and heart rate will be monitored continuously. The CPET is performed under medical supervision. The patient will be verbally encouraged in a standard manner. A CPET is safe for patients with cancer and we will adhere to the recommendations for testing procedure and safety [33]. Blood pressure will be measured with an automatic tonometer at rest and regularly during cycling. Also, an electrocardiogram (ECG) will be performed at rest and will be monitored continuously during the CPET.

#### Self-efficacy

Exercise-related self-efficacy will be assessed at baseline by six items, based upon SCT [29]. Items will be scored on a 5-point Likert scale with endpoints labelled “strongly disagree” and “strongly agree”.

#### Adherence and compliance

To monitor physical activity, patients are asked to keep a daily physical activity log to register the frequency and duration of activities they perform. They also register how many times the Jawbone UP2™ vibrates and their behaviour as the result of an alert. To determine adherence to the exercise intervention, attendance rate for the training sessions and compliance with the protocol (i.e. training intensity and duration according to protocol) will be recorded by the physiotherapist. Adherence to an active lifestyle will be monitored by the physiotherapist by two-weekly evaluations of the daily physical activity log.

### Statistical analyses

#### Sample size calculation

The sample size calculation is based on the intention-to-treat analyses of the primary effectiveness outcome QoL (EORTC-QLQ-30) [30]. Here, we determine a clinically relevant difference as a difference of 10 points [30]. A difference of 10 points is realistic because in a previous exercise trial in patients with cancer, QoL in the intervention group improved by 15.1 points (SD 17.7) and in the control group by 6.1 points (SD 17.1) using the EORTC-QLQ-30 after the 12-week intervention [34, 35]. Therefore, using the control group data from the previous trial and the 10-point difference, we assume a 6-point increase in QoL in the control group and a 16-point increase in the intervention group, among patients who accept the intervention in this cmRCT. We expect an attendance rate of 70% in the intervention group and assume that the improvement in non-attenders randomized to the intervention group (30%) is equal to the improvement in the control group (i.e. 6 points). Furthermore, we assume that non-attendance does not impact the standard deviation. As a result, we estimate a mean improvement of 13 points in the intervention group ((70*16 + 30*6)/100 = 13) instead of 16 points and a mean improvement of 6 points in the control group. Using these numbers, standard deviations of 17.7 and 17.1, power of 80% and alpha of 0.05, we calculated that 98 patients are needed in each group.

As we will use linear regression analyses adjusted for baseline, the correlation between baseline and follow up needs to be taken into account in the sample size calculation. Therefore, the calculated number of subjects should be multiplied by (1-*ρ* ^2^), plus one extra subject per group [36], where *ρ* represents the correlation between baseline and follow-up outcomes. In our previous trials [34, 35], we identified correlation of 0.4 between baseline and follow-up QoL. This leads to a final sample size of 83 patients per group (98*0.84 + 1). As recommended by Candlish [37], we will update the sample-size calculation before the end of the trial when the actual acceptance rate of the intervention deviates from the estimated rate and adapt the sample size accordingly.

#### Methodological outcomes

To assess contrasts in physical activity levels between the intervention and control group, we will compare changes in physical activity levels within the control group to changes in the intervention group using analysis of covariance (ANCOVA) or mixed linear regression models adjusted for baseline for the analyses of repeated outcomes [38].

Participation rate, intervention compliance, retention and drop-out will be compared to findings in previous conventional exercise-oncology RCTs. For instance, a systematic review reported median (IQR) rates of uptake, adherence and completion of exercise programmes in patients with cancer of 63% (33–80%), 84% (72–93%) and 87% (80–96%), respectively [39]. More recent and relevant publications, if available, will also be taken into account. Comparisons will be done by using the chi-squared test or independent samples *t* test.

#### Effectiveness outcomes

To assess differences in changes from baseline to 6 months in patient-related outcomes (QoL, fatigue, anxiety and depression and sedentary behaviour) between intervention and control, we will use intention-to-treat linear regression analyses adjusted for baseline. Mixed linear regression models adjusted for baseline will be used for the analyses of repeated outcomes (i.e. 6–24 months follow up).

The continuous within-group measurements in the intervention group, i.e. cardiorespiratory fitness, anthropometrics and patient-reported outcomes, will be analysed by the paired *t* test (if normally distributed) or Wilcoxon signed-rank test (if skewed).

From all UMBRELLA participants meeting the inclusion criteria for UMBRELLA Fit, we randomly selected patients for the intervention or control group with a 1:1 ratio. Besides comparison between patients in the intervention group and patients in the control group, we will investigate whether intervention effects change if we compare patients in the intervention group with all patients within UMBRELLA who are eligible for the UMBRELLA Fit study. Also, the timing of the regular measurements relative to the start and end of the intervention may vary between patients in the intervention group. Therefore, we will also assess QoL immediately at the start and end of the intervention. We will compare these outcome measures with the outcome measures from the regular UMBRELLA measurements completed before the start of the intervention and 6 months later (different and longer measurement intervals).

Patients may refuse the intervention after randomization and thus receive standard care. As a result, the estimated effect of the intervention may be diluted. We will conduct instrumental variable (IV) analyses [40, 41], taking non-compliance into account and assuming that the improvement in non-attenders randomized to the intervention group is equal to the improvement in the control group and that it does not impact the standard deviation. The IV method evaluates the effect of an intervention among “compliers”, who are individuals who would have received the intervention if it had been offered to them.

## Discussion

In the UMBRELLA Fit study, we will investigate the feasibility of the cmRCT design in the field of exercise-oncology. In addition, we will estimate the effectiveness of an exercise intervention on the QoL of inactive patients with breast cancer. Previous RCTs in the field of exercise-oncology have shown beneficial effects of physical activity on treatment-related side-effects, for example QoL. However, conventional RCTs in the field of exercise-oncology have several limitations, i.e. contamination, high drop-out in the control group, reduced generalizability, difficult accrual and insight only into the short-term effects. We hypothesized that the cmRCT design might overcome these shortcomings.

We acknowledge that the cmRCT design poses some challenges. A higher rate of drop-out after randomization is expected in the intervention group compared to conventional RCTs. Using intention-to-treat analysis, including all patients regardless of non-compliance and drop-out, might considerably dilute the actual treatment effect. Consequently, important clinical intervention effects might be missed. The estimated intervention effect depends on the proportion of non-compliance and becomes more diluted as non-compliance increases. Since compliance with the regular cohort measurements in the control group is expected to approximate 100% because they will not be informed about the study, the proportion of non-compliance in the control group will be lower when using the cmRCT design compared to a RCT. The lower non-compliance in the control group compensates the higher non-compliance in the intervention group [23].

The instrumental variable method will be used to estimate the intervention effect, taking drop-out/non-compliance after randomization into account. This method uses the random treatment assignment as an instrumental variable to control for non-compliance. However, disadvantage of this method in the cmRCT design is that we only know who will accept and complete the exercise programme in the intervention group, but this information is lacking from the control group. Patients who accept and complete the exercise programme might differ from those who do not. To deal with this lack of information, we will develop a prognostic model to estimate which patients would have been compliant in the control group by using the information on non-compliance of the intervention group.

Another challenge is the dependence on information collected within the cohort for the assessment of eligibility and for the outcome. If the available information is insufficient, there is a possibility that selected participants will not meet the inclusion criteria, e.g. due to severe comorbidity not yet registered in the cohort. However, since randomization has already been executed, these patients stay in the intention-to-treat analyses. In addition, another limitation of dependency on routine measurement outcomes is that the potential cmRCT outcomes are limited to outcome measures already collected in the observational cohort study. There is no opportunity to obtain additional information from the control group by additional measurements or at additional time points.

Patients are enrolled at 12 or 18 months after inclusion in UMBRELLA because at this time most of these patients have finished their primary treatment. In this period, the impact of diagnosis and treatment on normal daily living (e.g. complaints of fatigue, impaired quality of life) become more prominent [3–6]. Because previous research has shown that exercise has a beneficial effect on the QoL of patients with breast cancer in this period, the UMBRELLA Fit study gives the opportunity to assess the feasibility of the cmRCT design in the field of exercise-oncology by comparing the results of this trial with previous exercise trials in patients with breast cancer.

In summary, the UMBRELLA Fit study examines the effects of exercise on the QoL of patients with breast cancer, using the cmRCT design. The purpose of this study is twofold. The first goal is to determine the feasibility of the cmRCT design in the field of exercise-oncology and compare this with conventional RCTs in this field. The second goal is to examine the effectiveness of the exercise intervention on the QoL of patients with breast cancer in the short term (6 months) and long term (24 months).

### Trial status

Recruitment of participants is ongoing.

## Additional files

Additional file 1: The Standard Protocol Items: Recommendations for Interventional Trials (SPIRIT) 2013 checklist. (DOC 120 kb)

## Abbreviations

cmRCT: Cohort multiple randomized controlled trial
CPET: Cardiopulmonary exercise testing
EORTC QLQ-C30: European Organisation for Research and Treatment of Cancer Quality of Life Qestionnaire C30
HRR: Heart rate reserve
HR_rest_: Resting heart rate
IV: Instrumental variable
MET: Metabolic equivalent
PAR-Q: Physical Activity Readiness Questionnaire
RCT: Randomized controlled trial
RM: Repetition maximum
SCT: Social cognitive theory
SQUASH: Short QUestionnaire To ASsess Health-enhancing physical activity
UMBRELLA: Utrecht cohort for Multiple BReast cancer intErvention studies and Long-term evaLuAtion
UMC: University Medical Centre
VO2peak: Peak oxygen uptake

## References

[1] Ferlay J, Soerjomataram I, Ervik M, Dikshit R, Eser S, Mathers C, et al. Cancer incidence and mortality worldwide. In: IARC CancerBase No. 11. International Agency for Research on Cancer. 2013. http://gco.iarc.fr/today/home. Accessed 21 Oct 2016.

[2] Nederlandse Kankerregistratie (NKR). IKNL. http://www.cijfersoverkanker.nl. Accessed 21 Oct 2017.

[3] Servaes P, Verhagen C, Bleijenberg G. Fatigue in cancer patients during and after treatment. Eur J Cancer. 2002. https://doi.org/10.1016/S0959-8049(01)00332-X. 10.1016/s0959-8049(01)00332-x 11750837

[4] Stone P, Richards M, Hern R, Hardy J. A study to investigate the prevalence, severity and correlates of fatigue among patients with cancer in comparison with a control group of volunteers without cancer. Ann Oncol. 2000. https://doi.org/10.1023/A:1008331230608. 10.1023/a:1008331230608 10907949

[5] Arndt V, Stegmaier C, Ziegler H, Brenner H. A population-based study of the impact of specific symptoms on quality of life in women with breast cancer 1 year after diagnosis. Cancer. 2006. https://doi.org/10.1002/cncr.22274. 10.1002/cncr.22274 17048250

[6] Schmidt ME, Chang-Claude J, Vrieling A, Heinz J, Flesch-Janys D, Steindorf K. Fatigue and quality of life in breast cancer survivors: temporal courses and long-term pattern. J Cancer Surviv. 2012. https://doi.org/10.1007/s11764-011-0197-3. 10.1007/s11764-011-0197-3 22160661

[7] Fong DYT, Ho JWC, Hui BPH, Lee AM, Macfarlane DJ, Leung SSK, et al. Physical activity for cancer survivors: meta-analysis of randomised controlled trials. Br Med J. 2012. https://doi.org/10.1136/bmj.e70. 10.1136/bmj.e70 PMC3269661 22294757

[8] Jones LW, Alfano CM. Exercise-oncology research: Past, present, and future. Acta Oncol (Madr). 2013. https://doi.org/10.3109/0284186X.2012.742564. 10.3109/0284186X.2012.742564 23244677

[9] Cramp F, Byron-Daniel J. Exercise for the management of cancer-related fatigue. Cochrane Database Syst Rev. 2012. http://onlinelibrary.wiley.com/doi/10.1002/14651858.CD006145.pub3/full. 10.1002/14651858.CD006145.pub3 PMC8480137 23152233

[10] Buffart LM, Kalter J, Sweegers MG, Courneya KS, Newton RU, Aaronson NK, Jacobsen PB, May AM, Galvão DA, Chinapaw MJ, Steindorf K, Irwin ML, Stuiver MM, Hayes S, Griffith KA, Lucia A, Mesters I, van Weert E, Knoop H, Goedendorp MM, Mutrie N, Daley AJ, McCo BJ. Effects and moderators of exercise on quality of life and physical function in patients with cancer: an individual patient data meta-analysis of 34 RCTs. Cancer Treat Rev. 2017. https://doi.org/10.1016/j.ctrv.2016.11.010. 10.1016/j.ctrv.2016.11.010 28006694

[11] Lahart IM, Metsios GS, Nevill AM, Carmichael AR. Physical activity, risk of death and recurrence in breast cancer survivors: a systematic review and meta-analysis of epidemiological studies. Acta Oncol (Madr). 2015. https://doi.org/10.3109/0284186X.2014.998275. 10.3109/0284186X.2014.998275 25752971

[12] Phillips SM, Awick EA, Conroy DE, Pellegrini CA, Mailey EL, McAuley E. Objectively measured physical activity and sedentary behavior and quality of life indicators in survivors of breast cancer. Cancer. 2015. https://doi.org/10.1002/cncr.29620. 10.1002/cncr.29620 PMC4635035 26308157

[13] Kampshoff CS, van Mechelen W, Schep G, Nijziel MR, Witlox L, Bosman L, et al. Participation in and adherence to physical exercise after completion of primary cancer treatment. Int J Behav Nutr Phys Act. 2016. https://doi.org/10.1186/s12966-016-0425-3. 10.1186/s12966-016-0425-3 PMC5016937 27612561

[14] Trinh L, Amireault S, Lacombe J, Sabiston CM. Physical and psychological health among breast cancer survivors : interactions with sedentary behavior and physical activity. Psychooncology. 2015. https://doi.org/10.1002/pon.3872. 10.1002/pon.3872 29978929

[15] Thorpe KE, Zwarenstein M, Oxman AD, Treweek S, Furberg CD, Altman DG, et al. A pragmatic-explanatory continuum indicator summary (PRECIS): a tool to help trial designers. J Clin Epidemiol. 2009. https://doi.org/10.1016/j.jclinepi.2008.12.011. 10.1016/j.jclinepi.2008.12.011 19348971

[16] Steins Bisschop CN, Courneya KS, Velthuis MJ, Monninkhof EM, Jones LW, Friedenreich C, et al. Control group design, contamination and drop-out in exercise oncology trials: a systematic review. PLoS One. 2015. https://doi.org/10.1371/journal.pone.0120996. 10.1371/journal.pone.0120996 PMC4376879 25815479

[17] Courneya KS, Forbes CC, Trinh L, Sellar CM, Friedenreich CM, Reiman T. Patient satisfaction with participation in a randomized exercise trial: effects of randomization and a usual care posttrial exercise program. Clin Trials. 2013. https://doi.org/10.1177/1740774513495985. 10.1177/1740774513495985 23918843

[18] Gollhofer SM, Wiskemann J, Schmidt ME, Klassen O, Ulrich CM, Oelmann J, et al. Factors influencing participation in a randomized controlled resistance exercise intervention study in breast cancer patients during radiotherapy. BMC Cancer. 2015. https://doi.org/10.1186/s12885-015-1213-1. 10.1186/s12885-015-1213-1 PMC4466838 25885634

[19] Relton C, Torgerson D, O’Cathain A, Nicholl J. Rethinking pragmatic randomised controlled trials: introducing the “cohort multiple randomised controlled trial” design. BMJ. 2010. https://doi.org/10.1136/bmj.c1066. 10.1136/bmj.c1066 20304934

[20] Young-Afat DA, Verkooijen HM, van Gils CH, van der Velden JM, Burbach JP, Elias SG, et al. Staged-informed consent in the cohort multiple randomized controlled trial design. Epidemiology. 2016. https://doi.org/10.1097/EDE.0000000000000435. 10.1097/EDE.0000000000000435 27035689

[21] Velthuis MJ, May AM, Monninkhof EM, van der Wall E, Peeters PH. Alternatives for randomization in lifestyle intervention studies in cancer patients were not better than conventional randomization. J Clin Epidemiol. 2012. https://doi.org/10.1016/j.jclinepi.2011.03.015. 10.1016/j.jclinepi.2011.03.015 21856119

[22] Travier N, Velthuis MJ, Steins Bisschop CN, van den Buijs B, Monninkhof EM, Backx F, et al. Effects of an 18-week exercise programme started early during breast cancer treatment: a randomised controlled trial. BMC Med. 2015. https://doi.org/10.1186/s12916-015-0362-z. 10.1186/s12916-015-0362-z PMC4461906 26050790

[23] Van der Velden JM, Verkooijen HM, Young-Afat DA, Burbach JPM, van Vulpen M, Relton C, et al. The cohort multiple Randomized Controlled Trial design: a valid and efficient alternative to pragmatic trials? Int J Epidemiol. 2016. https://doi.org/10.1093/ije/dyw050. 10.1093/ije/dyw050 27118559

[24] Van Waart H, van Harten WH, Buffart LM, Sonke GS, Stuiver MM, Aaronson NK. Why do patients choose (not) to participate in an exercise trial during adjuvant chemotherapy for breast cancer? Psychooncology. 2016. https://doi.org/10.1002/pon.3936. 10.1002/pon.3936 26282696

[25] Young-Afat DA, van Gils CH, van den Bongard DHJ, Verkooijen HM, on behalf of the UMBRELLA study groups. Cohort profile: The Utrecht cohort for Multiple BREast cancer intervention studies and Long-term evaLuAtion (UMBRELLA). Submitted. 10.1007/s10549-017-4242-4 PMC5487711 28444532

[26] Wendel-Vos GC, Schuit A, Saris WHM, Kromhout D. Reproducibility and relative validity of the short questionnaire to assess health-enhancing physical activity. J Clin Epidemiol. 2003. https://doi.org/10.1016/S0895-4356(03)00220-8. 10.1016/s0895-4356(03)00220-8 14680666

[27] Thomas S, Reading J, Shephard R. Revision of the Physical Activity Readiness Questionnaire (PARQ). Can J Sport Sci. 1992;17(4):338–45. 1330274

[28] Garber CE, Blissmer B, Deschenes MR, Franklin BA, Lamonte MJ, Lee IM, et al. Quantity and quality of exercise for developing and maintaining cardiorespiratory, musculoskeletal, and neuromotor fitness in apparently healthy adults: Guidance for prescribing exercise. Med Sci Sports Exerc. 2011; https://doi.org/10.1249/MSS.0b013e318213fefb. 10.1249/MSS.0b013e318213fefb 21694556

[29] Bandura A (1986). Social foundations of thought and action: a social cognitive theory.

[30] Aaronson NK, Ahmedzai S, Bergman B, Bullinger M, Cull A, Duez NJ, et al. The European Organization for Research and Treatment of Cancer QLQ-C30: a quality-of-life instrument for use in international clinical trials in oncology. J Natl Cancer Inst. 1993. https://doi.org/10.1093/jnci/85.5.365. 10.1093/jnci/85.5.365 8433390

[31] Craig CL, Marshall AL, Sjöström M, Bauman AE, Booth ML, Ainsworth BE, et al. International physical activity questionnaire: 12-country reliability and validity. Med Sci Sports Exerc. 2003. https://doi.org/10.1249/01.MSS.0000078924.61453.FB. 10.1249/01.MSS.0000078924.61453.FB 12900694

[32] Osborne RH, Elsworth GR, Sprangers MAG, Oort FJ, Hopper JL. The value of the Hospital Anxiety and Depression Scale (HADS) for comparing women with early onset breast cancer with population-based reference women. Qual Life Res. 2004. https://doi.org/10.1023/B:QURE.0000015292.56268.e7. 10.1023/B:QURE.0000015292.56268.e7 15058800

[33] Jones LW, Eves ND, Peppercorn J. Pre-exercise screening and prescription guidelines for cancer patients. Lancet Oncol. 2010. https://doi.org/10.1016/S1470-2045(10)70184-4. 10.1016/S1470-2045(10)70184-4 PMC3615547 20708967

[34] Young-Afat DA, van Gils CH, van den Bongard DHJ, Verkooijen HM, on behalf of the UMBRELLA study groups. The Utrecht cohort for Multiple BREast cancer intervention studies and Long-term evaLuAtion (UMBRELLA): objectives, design, and baseline results. Breast Cancer Res Treat. 2017. doi:10.1007/s10549-017-4242-4. 10.1007/s10549-017-4242-4 PMC5487711 28444532

[35] May AM, Korstjens I, van Weert E, van den Borne B, Hoekstra-Weebers JEHM, van der Schans CP, et al. Long-term effects on cancer survivors’ quality of life of physical training versus physical training combined with cognitive-behavioral therapy: results from a randomized trial. Support Care Cancer. 2009. https://doi.org/10.1007/s00520-008-0519-9. 10.1007/s00520-008-0519-9 18953578

[36] Borm GF, Fransen J, Lemmens WAJG. A simple sample size formula for analysis of covariance in randomized clinical trials. J Clin Epidemiol. 2007. https://doi.org/10.1016/j.jclinepi.2007.02.006. 10.1016/j.jclinepi.2007.02.006 17998077

[37] Candlish J, Pate A, Sperrin M, van Staa T, Package W. Evaluation of biases present in the cohort multiple randomised controlled trial design : a simulation study. BMC Med Res Methodol. 2017. https://doi.org/10.1186/s12874-017-0295-7. 10.1186/s12874-017-0295-7 PMC5282910 28143408

[38] Vickers AJ, Altman DG. Analysing controlled trials with baseline and follow up measurements. 2001. https://doi.org/10.1136/bmj.323.7321.1123. 10.1136/bmj.323.7321.1123 PMC1121605 11701584

[39] Maddocks M, Mockett S, Wilcock A. Is exercise an acceptable and practical therapy for people with or cured of cancer? A systematic review. Cancer Treat Rev. 2009. https://doi.org/10.1016/j.ctrv.2008.11.008. 10.1016/j.ctrv.2008.11.008 19131171

[40] Hertogh EM, Schuit JA, Peeters PHM, Monninkhof EM. Noncompliance in lifestyle intervention studies: the instrumental variable method provides insight into the bias. J Clin Epidemiol. 2010. https://doi.org/10.1016/j.jclinepi.2009.10.007. 10.1016/j.jclinepi.2009.10.007 20189770

[41] Hewitt CE, Torgerson DJ, Miles JNV. Is there another way to take account of noncompliance in randomized controlled trials? CMAJ. 2006. https://doi.org/10.1503/cmaj.051625. 10.1503/cmaj.051625 PMC1534095 16908892

